# Effect of polyglycerol polyricinoleate on the inhibitory mechanism of sesamol during bulk oil oxidation

**DOI:** 10.1038/s41598-022-16201-7

**Published:** 2022-07-13

**Authors:** Malihe Keramat, Mohammad-Taghi Golmakani, Mehrdad Niakousari

**Affiliations:** Department of Food Science and Technology, School of Agriculture, Shiraz University, Shiraz, 7144165186 Iran

**Keywords:** Chemical biology, Health care, Chemistry

## Abstract

In this study, effects of sesamol on improving the oxidative stability of sunflower oil and its oil-in-water emulsion was investigated. To investigate the kinetic parameters related to the initiation and propagation stages of oxidation, a sigmoidal-model was used. Sesamol exhibited higher antioxidant activity in sunflower oil-in-water emulsion than that of sunflower oil. In both sunflower oil and sunflower oil-in-water emulsion, the inhibitory effect of sesamol against lipid oxidation continued even after the induction period. To improve the efficiency of sesamol in sunflower oil, polyglycerol polyricinoleate (PGPR) was incorporated into the functional environment of the sesamol. Sesamol exhibited a synergistic effect with PGPR during both initiation (synergistic effect of 68.87%) and propagation (synergistic effect of 36.84%) stages. Comparison of the size of reverse micelles in samples containing PGPR with those without PGPR revealed that PGPR can enhance the efficiency of sesamol by increasing the acceptance capacity of lipid hydroperoxides in reveres micelles structures. This can result in enhancing the effective collisions between sesamol and lipid hydroperoxides in the presence of PGPR. The water produced as a major byproduct of oxidation played a key role on the antioxidant activity of sesamol alone or in combination with PGPR during oxidation process.

## Introduction

Lipid oxidation is a complex phenomenon that causes the loss of quality in lipid-containing foods^1^. Lipid oxidation in bulk oil and oil-in-water emulsion is primarily an interfacial phenomenon. In bulk oils, surface active agents can self-assemble in the presence of water to form association colloids such as reverse micelles and lamellar bilayers^2^. Reverse micelles are dynamic objects that are dispersed according to Brownian motion and influence the partitioning of compounds in bulk oils and trap polar or hydrophilic molecules in their structure. Therefore, these structures play a crucial role in the lipid oxidation pathways in bulk oils. Lipid oxidation mechanisms in bulk oils actually take place inside-or in the immediate vicinity-of reverse micelles. Physical properties (size, shape, and concentration) of reverse micelles, their chemical nature, their capacity to solubilize and interact with different components from antioxidants to pro-oxidants, and the capacity of these components to concentrate at the water–oil interface of the reverse micelles can affect lipid oxidation in bulk oils^3^.

The initial step in lipid oxidation of oil-in-water emulsion has been suggested to take place at the surface of oil droplets, where unsaturated fatty acids in the oil phase and transition metal ions in the water phase come into close proximity^4^. The oxidative stability of oil-in-water emulsions is influenced by a number of factors including size of the oil droplets, physical and chemical characteristics of the interfacial area, pH, and components present in the oil and aqueous phases^5^. Another important factor which is often ignored in determining the lipid oxidation rate is transport of lipid substrates, transition metal ions, free radicals, and antioxidants in oil-in-water emulsion. The mechanisms of this mass transport occur through three pathways of diffusion, collision-exchange-separation, or micelle-assisted transfer. Water soluble compounds are hypothesized to be transferred between oil droplets through the diffusion pathway in the water phase of oil-in-water emulsion. Hydrophobic compounds are hypothesized to be transferred either by the collision of adjacent droplets or by micelle-assisted mechanisms. The transfer rate is higher through the micelle-assisted pathway and depends on the concentration and size of micelles^6^.

Sesamol (3,4-(methylenedioxy) phenol) is a relatively lipophilic monophenol, which originates from sesame oil. This compound has a powerful antioxidant activity and is often used as an antioxidant in food and medicine^7,8^. Sesamol can efficiently scavenges hydroxyl, lipid peroxyl, one-electron oxidizing, tryptophanyl, and organo-haloperoxyl radicals^9^. The efficiency of a selected antioxidant in the oil-based food products is not only determined by its chemical reactivity. Antioxidant activity of an antioxidant in oil-based food products is the result of more complex reactions and phenomena. Many factors such as chemical reactivity and interaction of an antioxidant with other food compounds, environmental conditions, and interfacial activity of the antioxidant compound can affect its efficiency in oil-based food products^2,10^. A highly reactive antioxidant needs to be located at water–oil interface of reverse micelles in bulk oil and at the surface of oil droplets in oil-in-water emulsion to encounter pro-oxidants and protect unsaturated fatty acids^11^.

Incorporating surfactants into bulk oils at concentrations below their critical micelle concentration (CMC) can improve the interfacial activity of antioxidants. Considering the fact that surfactants can significantly reduce interfacial tension, the number and size of the reverse micelles are likely to increase significantly in the presence of surfactants. As a result, there can be an increase in the acceptance capacity of LOOHs in these structures. Accordingly, more interactions can occur between antioxidant molecules and LOOHs in the presence of surfactants. In addition, increasing the number of reverse micelles in the presence of surfactants can excite the movement of the nonpolar part of antioxidant molecules into the water–oil interface of reverse micelles^12,13^.

In this study, effects of sesamol on inhibiting the oxidation of sunflower oil and its oil-in-water emulsion was investigated. A sigmoidal kinetic model was used to evaluate the inhibitory effect of sesamol over the whole practical range of peroxidation, including the initiation and propagation stages. In addition, polyglycerol polyricinoleate (PGPR) which is considered as a surfactant with low HLB (hydrophilic lipophilic balance) value was incorporated into sunflower oil to improve the antioxidant activity of sesamol (based on the interfacial phenomena), so as to inhibit the oxidation of stripped sunflower oil. Furthermore, various kinetic parameters and rate constants arising from the initiation and propagation stages of lipid oxidation were evaluated to elucidate the details of physicochemical events that occurred during the oxidation process. This can be considered as the first attempt to use these evaluations in describing how PGPR can affect the interfacial activity of sesamol during the initiation and propagation stages of oxidation.

## Materials and methods

### Materials

Commercial sunflower oil was purchased from a local market. Sesamol (> 98%), Tween 80, ammonium thiocyanate, potassium dihydrogen phosphate, di-potassium hydrogen phosphate, PGPR, and barium chloride were purchased from Sigma-Aldrich Company (St. Louis, MO). Chloroform, *n*-heptane, hydrochloric acid, and methanol were purchased from Merck Company (Darmstadt, Germany).

### Sunflower oil stripping

Sunflower oil stripping was performed by an adsorption chromatography column. A glass column (36 cm length and 2.9 cm internal diameter) was packed with silica gel (20.04 g) and aluminum oxide 60 (140.04 g). Silica gel and aluminum oxide 60 were activated at 180 °C for 4 h. Sunflower oil (120 g) was passed through the column by a vacuum pump. The stripping procedure was performed twice to gain inconsiderable levels of indigenous antioxidative compounds and lipid hydroperoxides^14^.

### Preparation of sunflower oil samples

Sesamol was dissolved in acetone and added to the purified sunflower oil at concentration of 0.05% (w/w) oil. Then, acetone was evaporated under a stream of nitrogen. To provide samples containing PGPR, 0.05% (w/w oil) of PGPR was dissolved in ethyl acetate (1:10 w/v) for 1 h at 40 °C by a magnetic thermo-stirrer. Then, purified sunflower oil was slowly added to the cooled solution and the stirring process remained at ambient temperature for 10 min. Afterwards, ethyl acetate was eliminated by a rotary evaporator. In the next step, sesamol (0.05% w/w oil) was separately added to the purified sunflower oil containing PGPR. The CMC value of PGPR vary between 0.76 and 1.50% in the oil phase^15^. Since surfactants self-aggregate and form reverse micelles above their CMC value^16^, a concentration lower than the CMC of PGPR was used in this study.

### Preparation of sunflower oil-in-water emulsion samples

Sunflower oil-in-water emulsion samples were prepared using the emulsion phase inversion method. Initially, purified sunflower oil and Tween 80 were mixed using a magnetic stirrer (750 rpm) for 30 min. Then, sesamol (0.5%, w/w oil) was dissolved in acetone and added to the purified sunflower oil. After that, the acetone was removed from purified sunflower oil samples using nitrogen stream. Finally, potassium phosphate buffer solution (0.04 mol L^−1^, pH  7) was titrated into purified sunflower oil containing Tween 80 with a flow rate of 300 µL/min, while continuing to stir the system by magnetic stirrer (750 rpm). The Tween 80:oil ratio was 1:1 and the oil:water ratio was 1:10^17^. The particle size of the sunflower oil-in-water emulsion was 181.05 ± 0.49 nm.

### Monitoring accumulation of LOOHs

Accumulating of LOOHs during storage at 55 °C was monitored by measuring peroxide value (PV) at certain time intervals. To determine PV, the oil samples (0.001–0.3 g) were mixed with 9.8 mL chloroform–methanol (7:3, v/v) using a vortex mixer for 2–4 s. Then, 50 μL of ammonium thiocyanate aqueous solution (30%, w/v) was added to the oil sample and shaked for 5 s. After that, 50 μL Iron (II) chloride solution ([0.25 g FeSO4.7H_2_O dissolved in 25 mL H_2_O] + [0.2 g barium chloride dehydrate dissolved in 25 mL H_2_O] + 1 mL HCl 10 N, and then the resultant solution was filtered to remove barium sulphate deposits) was added. After 5 min incubation at room temperature, absorption values of samples were determined at 500 nm^18^. For oil extraction from emulsions, 1.5 mL of chloroform:methanol (1:1, v/v) was blended with 0.3 mL emulsion and vortexed for 1 min. Then, the mixture was centrifuged for 5 min at 1300*g*. The lower lipid layer was collected and its solvent evaporated using nitrogen stream.

### Kinetic study

Kinetic curves of LOOHs accumulation were drawn by plotting the changes in PV (meq kg^-1^) versus time.

LOOHs concentration linearly increased during induction period (IP) according to Eq. (1).

1 $$ {\text{[LOOH] = k}}_{{\text{i}}} {\text{(t) + }}\left[ {{\text{LOOH}}} \right]_{{0}} $$

where [LOOH]_0_ (meq kg^−1^) is lipid hydroperoxide concentration at t = 0.

The pseudo-zero order rate constant of the initiation stage (k_i_, meq kg^−1^ h^−1^) was expressed by Eq. (2):

2 $$ \frac{{\text{d[LOOH]}}}{{{\text{dt}}}}{\text{ = k}}_{{\text{i}}} $$

**Figure 1:**
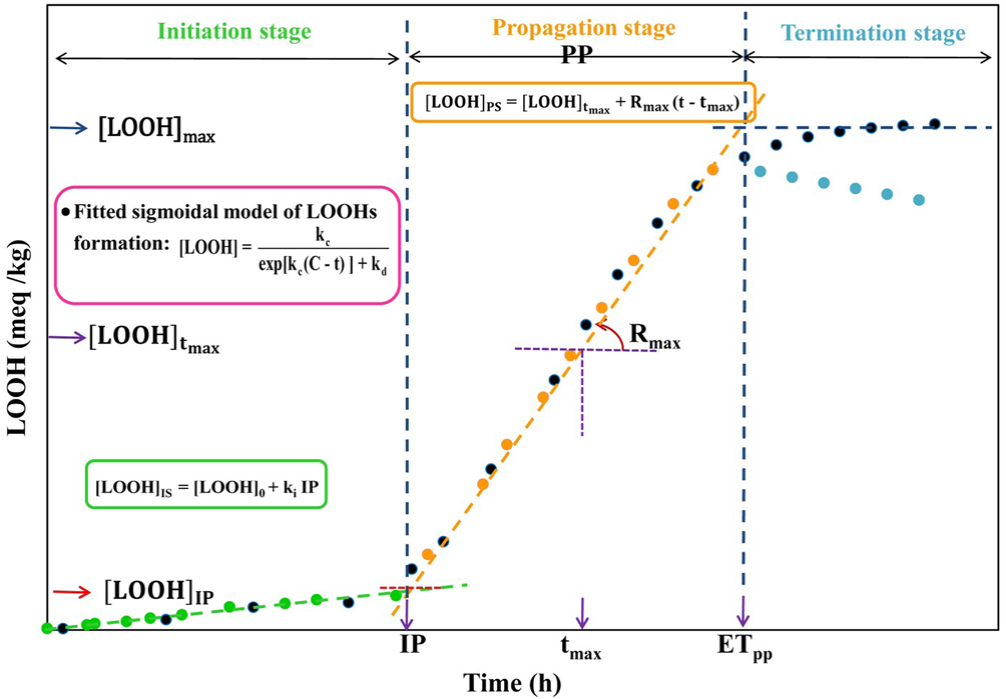
Schematic curve of lipid hydroperoxides (LOOHs) production and a guide of calculated kinetic parameters. *IP* induction period, *PP* duration of the propagation stage, *ET*_*pp*_ end time of propagation stage, *ki* pseudo-zero order rate constant at the initiation stage, *k*_*c*_ pseudo-first order rate constant of LOOHs formation at the propagation stage, *k*_*d*_ pseudo-second order rate constant of LOOHs decomposition at the propagation stage, *R*_*max*_ maximum rate of LOOHs formation in the propagation stage, [*LOOH*]_0_ LOOH concentration at t = 0, [*LOOH*]_IP_ LOOHs concentration at IP point, [*LOOH*]_*Tmax*_ LOOHs concentration at the point of the maximum rate of LOOHs formation, [*LOOH*]_*max*_ maximum concentration of produced LOOHs, *T*_*max*_ occurrence time of maximum rate of LOOHs formation, *C* integration constant^20^.

The increase pattern of [LOOH] concentration over the whole range of peroxidation, including the initiation and propagation stages was expressed by Eq. (3).

3 $$ {\text{[LOOH] = }}\frac{{{\text{k}}_{{\text{c}}} }}{{{\text{exp[k}}_{{\text{c}}} {\text{(C - t)] + k}}_{{\text{d}}} }} $$

where k_c_ (h^−1^) is the pseudo-first order rate constant of LOOHs formation at the propagation stage, k_d_ (kg meq^−1^ h^−1^) is the pseudo-second order rate constant of LOOHs decomposition at the propagation stage, C (kg meq^−1^) is an overall integration constant^19^. The Eq. (3) exhibits a sigmoidal characteristic as illustrated in Fig. 1.

Maximum concentration of produced LOOHs ([LOOH]_max_, meq kg^−1^) was determined according to Eq. (4).

4 $$ \left[ {{\text{LOOH}}} \right]_{{{\text{max}}}} \, = \,{\text{lim }}t \to \infty \;\left\{ {\frac{{{\text{k}}_{{\text{c}}} }}{{{\text{exp}}\left[ {{\text{k}}_{{\text{c}}} {\text{(C - t) + kd}}} \right]}}} \right\} _{ = } \frac{{{\text{k}}_{{\text{c}}} }}{{{\text{k}}_{{\text{d}}} }} $$

The second derivative of the sigmoidal equation (d^2^[LOOH]/dt^2^) at t = 0 provided the coordinates of a turning point (T_max_, h). In this point, the rate of LOOH accumulation approaches a maximum value (R_max_, meq kg^-1^ h^−1^) in the propagation stage (Fig. 1). T_max_ was calculated according to Eq. (5).

5 $$ {\text{T}}_{{{\text{max}}}} { = }\frac{{{\text{k}}_{{\text{c}}} {\text{C - lnk}}_{{\text{d}}} }}{{{\text{k}}_{{\text{c}}} }} $$

LOOHs concentration at the point of the maximum rate of LOOHs formation ([LOOH]_Tmax_, meq kg^-1^) was calculated according to Eq. (6).

6 $$ \left[ {{\text{LOOH}}} \right]_{{{\text{Tmax}}}} { = }\frac{{{\text{k}}_{{\text{c}}} }}{{{\text{2k}}_{{\text{d}}} }} $$

R_max_ was calculated using Eq. (7).

7 $$ {\text{R}}_{{{\text{max}}}} { = }\left( {\frac{{\text{d[LOOH]}}}{{{\text{dt}}}}} \right)_{{{\text{max}}}} { = }\frac{{{\text{k}}^{{2}}_{{\text{c}}} }}{{{\text{4k}}_{{\text{d}}} }} $$

The propagation oxidizability parameter (R_n_, h^−1^) was calculated using Eq. (8).

8 $$ {\text{R}}_{{\text{n}}} { = }\frac{{{\text{R}}_{{{\text{max}}}} }}{{\left[ {{\text{LOOH}}} \right]_{{{\text{max}}}} }} $$

The parameters IP (h) and [LOOH]_IP_ (meq kg^−1^) were calculated according to Eqs. (9) and (10), respectively^20^.

9 $$ {\text{IP = }}\frac{{{\text{k}}_{{\text{c}}} {\text{(2 - k}}_{{\text{c}}} {\text{C + lnk}}_{{\text{d}}} {) - 4}\left[ {{\text{LOOH}}} \right]_{{0}} {\text{k}}_{{\text{d}}} }}{{{\text{4k}}_{{\text{i}}} {\text{k}}_{{\text{d}}} {\text{ - k}}^{{2}}_{{\text{c}}} }} $$

10 $$ \left[ {{\text{LOOH}}} \right]_{{{\text{IP}}}} {\text{ = k}}_{{\text{i}}} {\text{(IP) + }}\left[ {{\text{LOOH}}} \right]_{{0}} $$

The initiation oxidizability parameter (O_i_, h^2^ meq^−1^ kg), which unifies k_i_ and IP, could show well the resistance of the sunflower oil samples to the formation of LOOH during the initiation stage. The O_i_ was calculated using Eq. (11).

11 $$ {\text{O}}_{{\text{i}}} { = }\frac{{{\text{IP}}}}{{{\text{k}}_{{\text{i}}} }} $$

Antioxidant effectiveness in the initiation stage was calculated using Eq. (12).

12 $$ {\text{E}}_{{\text{i}}} { = }\frac{{{\text{IP}}_{{{\text{AH}}}} }}{{{\text{IP}}_{{\text{C}}} }} $$

where IP_AH_ is the IP in the presence of antioxidant and IP_C_ is the IP in the absence of antioxidant.

Oxidation rate ratio (ORR) in the initiation stage, which is an inverse measure of antioxidant strength, was calculated using Eq. (13).

13 $$ {\text{ORR}}_{{\text{i}}} { = }\frac{{{\text{k}}_{{\text{IP, A}}} }}{{{\text{k}}_{{\text{IP, B}}} }} $$

where k_i, AH_ is the value of k_i_ in the presence of antioxidant and k_i, C_ is the value of k_i_ in the absence of antioxidant. Antioxidant activity (A) was calculated using Eq. (14).

14 $$ {\text{A  }}= \frac{{{\text{F}}_{{\text{i}}} }}{{{\text{ORR}}_{{\text{i,}}} }}{ = }\frac{{{\text{O}}_{{\text{i, A}}} }}{{{\text{O}}_{{\text{i, B }}} }} $$

Synergistic effect of sesamol with PGPR during the initiation stage (SE_i_) was calculated according to Eq. (15).

15 $$ {\text{SE}}_{{\text{i}}} { = (1 - }\frac{{{\text{IP}}_{{\text{i, AH}}} + {\text{  IP}}_{{\text{i, P}}} - {\text{  2IP}}_{{\text{i, C}}} }}{{{\text{2(IP}}_{{\text{i, AH }}}+ {\rm P} - {\text{  IP}}_{{\text{i, C}}} {)}}}{{) \times 100}} $$

where IP_i,AH_, IP_i,P_, IP_i_,_C,_ and IP_i, AH+P_ are initiation oxidizability parameter of the antioxidant per se, PGPR per se, control, and antioxidant + PGPR, respectively.

The end time of the propagation stage (ET_pp_, h) was calculated according to Eq. (16).

16 $$ {\text{ET}}_{{{\text{pp}}}} { = }\frac{{{\text{4k}}_{{\text{d}}} {\text{R}}_{{{\text{max}}}} {\text{ - k}}_{{\text{c}}} {\text{R}}_{{\text{n}}} {\text{(2 - k}}_{{\text{c}}} {\text{C + lnk}}_{{\text{d}}} {)}}}{{{\text{4k}}_{{\text{d}}} {\text{R}}_{{{\text{max}}}} {\text{R}}_{{\text{n}}} }} $$

Propagation period (PP, h) was calculated according to Eq. (17).

17 $$ {\text{PP}}\, = \,{\text{ET}}_{{{\text{pp}}}} {-}{\text{IP}} $$

Antioxidant effectiveness during the propagation stage was calculated using Eq. (18).

18 $$ {\text{E}}_{{\text{p}}} { = }\frac{{{\text{PP}}_{{{\text{AH}}}} }}{{{\text{PP}}_{{\text{C}}} }} $$

where PP_AH_ is the PP in the presence of antioxidant and PP_C_ is the PP in the absence of antioxidant.

The oxidation rate ratio of LOOHs formation during the propagation stage was calculated using Eq. (19).

19 $$ {\text{ORR}}_{{\text{c}}} { = }\frac{{{\text{k}}_{{\text{c,AH}}} }}{{{\text{k}}_{{\text{c,C}}} }} $$

where k_c, AH_ is the value of k_c_ in the presence of antioxidant and k_c, C_ is the value of k_c_ in the absence of antioxidant.

The oxidation rate ratio of LOOHs decomposition during the propagation stage was calculated using Eq. (20).

20 $$ {\text{ORR}}_{{\text{d}}} { = }\frac{{{\text{k}}_{{\text{d,AH}}} }}{{{\text{k}}_{{\text{d,C}}} }} $$

where k_d, AH_ is the value of k_d_ in the presence of antioxidant and k_d, C_ is the value of k_d_ in the absence of antioxidant.

IA_c_ that is the inhibitory activity against the LOOHs formation, and IA_d_ that is the inhibitory activity against the LOOHs decomposition were calculated using Eqs. (21) and (22), respectively^21^.

21 $$ {\text{IA}}_{{\text{c}}} { = }\frac{{{\text{E}}_{{\text{p}}} }}{{{\text{ORR}}_{{\text{c}}} }} $$

22 $$ {\text{IA}}_{{\text{d}}} { = }\frac{{{\text{E}}_{{\text{p}}} }}{{{\text{ORR}}_{{\text{d}}} }} $$

Synergistic effect of sesamol with PGPR during the propagation stage (SE_p_) was calculated according to Eq. (23):

23 $$ {\text{SE}}_{{\text{p}}} { = (1 - }\frac{{{\text{T}}_{{{\text{max}}}} +{\text{  T}}_{{\text{max, P}}} - {\text{  2T}}_{{\text{max, C}}} }}{{{\text{2(T}}_{{{{\rm max, AH} + {\rm P}}}} -{\text{  T}}_{{\text{max, C}}} {)}}}{{) \times 100}} $$

where T_max,AH_, T_max,P_, T_max_,_C,_ and T_max, AH+P_ are propagation oxidizability parameter of the antioxidant per se, PGPR per se, control, and antioxidant + PGPR, respectively.

### Water content

Changes in water content of sunflower oil samples during lipid oxidation were determined by coulometric Karl Fischer titrator (KF Titrino, Metrohm, Herisau, Switzerland)) using the ASTM E1064 standard test method^22^.

### Particle size

Particle size of sunflower oil and sunflower oil-in-water emulsion samples were determined using dynamic light scattering instrument (SZ-100 nanopartica series, Horiba Ltd., Kyoto, Japan) at light scattering angle of 173°. Sunflower oil-in-water emulsion was diluted 100-times using potassium phosphate buffer (0.04 mol L^−1^, pH  7) prior to analysis to avoid multiple scattering effects.

### Viscosity

The viscosity of sunflower oil and its oil-in-water emulsion was measured using a capillary viscometer (Schott Gerate 51810; Germany). The dynamic viscosity was calculate at 25 °C using Eq. (24)^23^.

24 $$ {\text{Dynamic viscosity }}\left( {\text{mPa s}} \right) \, = {\text{ density }}\left( {{\text{kg m}}^{{ - {3}}} } \right) \, \times {\text{ kinematic viscosity }}\left( {{\text{mm}}^{{2}} {\text{s}}^{{ - {1}}} } \right) \, \times { 1}0^{{ - {3}}} $$

### Statistical analysis

All experiments were done in three independent tests. Significant differences among the mean values were measured using a one-way analysis of variance. Comparisons of the mean values were carried out using Duncan’s multiple range test (*P* < 0.05). Statistical and regression analyses were performed using SPSS, CurveExpert, and Microsoft Office Excel software.

## Results and discussion

### Evaluating kinetic parameters related to the initiation stage of lipid oxidation

**Figure 2:**
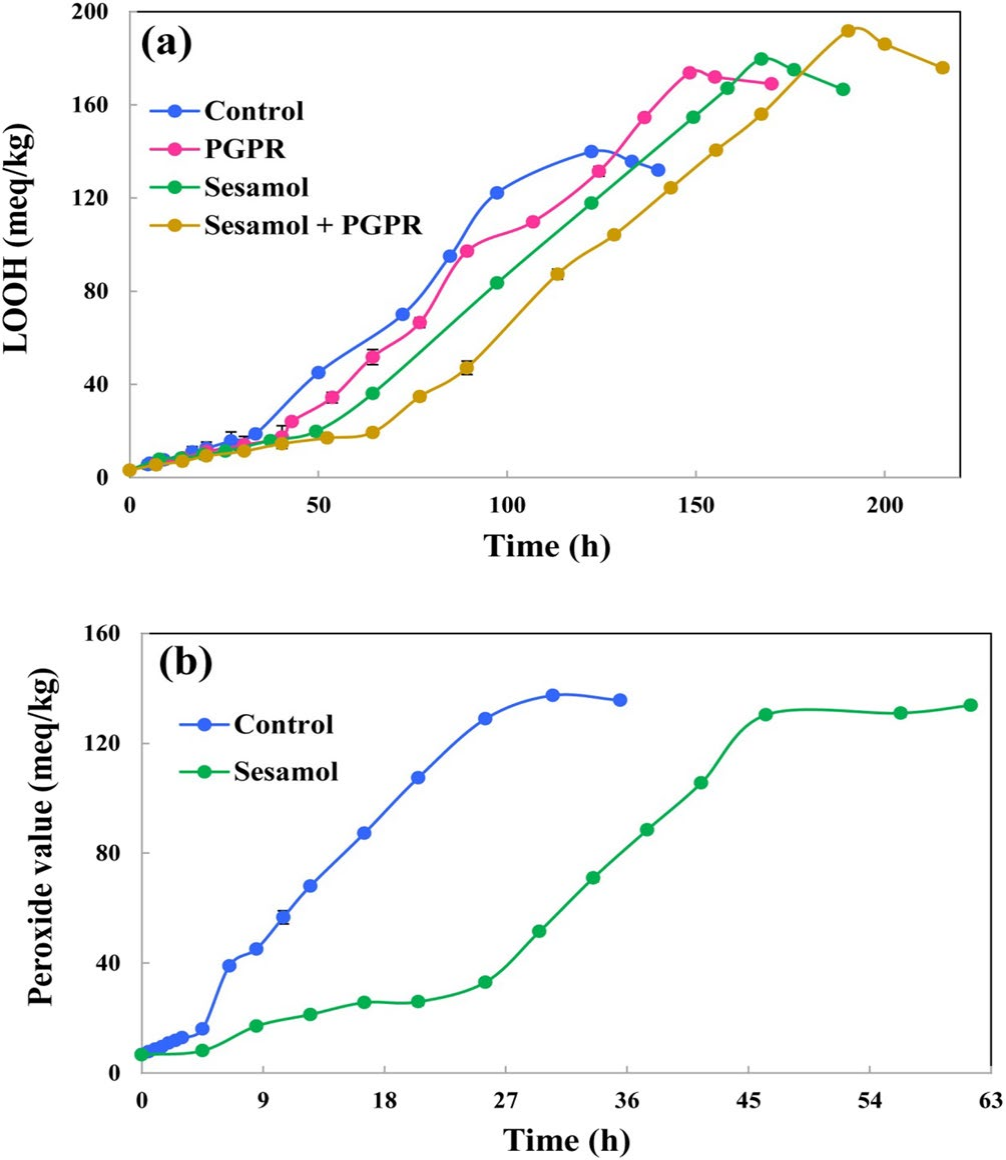
Sigmoidal curve of lipid hydroperoxides (LOOH) accumulation in peroxidation of (**a**) sunflower oil and (**b**) sunflower oil-in-water emulsion samples at 55 °C. *PGPR* polyglycerol polyricinoleate.

The predicted sigmoidal kinetic model fitted well on the curve of LOOHs production (R^2^ ≥ 0.985) and distinguished the different stages of the oxidation process (Fig. 2). During lipid oxidation process, a variety of free radicals with different redox potentials (Eh) such as alkoxyl (LO^·^: 1600 mV), hydroxyl (^·^OH: 2320 mV), peroxyl (LOO^·^: 1000 mV), and alkyl (L^·^: 600 mV) can be produced in the lipid systems^24,25^. At the beginning of the oxidation process, the only pathway of LOOHs production is the conversion of L^·^ to LOO^·^ (due to its low Eh) and its attack on the hydrogen attached to allylic or bis-allylic carbon^26^. The initiation stage can be basically characterized by IP, k_i_, and [LOOH]_IP_. In both sunflower oil and its oil-in-water emulsion, sesamol significantly increased the IP, E_i_, and A values and reduced the ORR and O_i_ values, compared to the control sample. Also, in both sunflower oil and sunflower oil-in-water emulsion, the [LOOH]_IP_ value of sample containing sesamol was higher than the control sample (Table 1). This indicates that sesamol has participated in chain termination reaction (AH + LOO^·^ → LOOH + A^·^) and increased hydroperoxide concentration. Sesamol exhibited higher efficiency in increasing the A value of sunflower oil-in-water emulsion than the sunflower oil. The location of antioxidant molecules or, indeed, their interfacial distribution in lipid system is an important parameter that can affect their efficiency on inhibiting the lipid oxidation. In sunflower oil, hydrophilic antioxidants that are able to locate at the interface of the reverse micelles can inhibit the lipid oxidation more efficiently than hydrophobic antioxidants, while in sunflower oil-in-water emulsion, hydrophobic antioxidants that are able to locate at the surface of the oil droplets are more effective than hydrophilic antioxidants^11^. Sesamol is a moderately lipophilic compound that has low solubility in water (~ 38.8 mg mL^−1^). The partition coefficient (log P) value of sesamol is 1.29. The log P value is positive if a substance is more soluble in fat-like solvents, and is negative if it is more soluble in water^27^. Accordingly, sesamol has higher affinity for locating at the surface of oil droplets of sunflower oil-in-water emulsion than locating at the interface of reverse micelles of sunflower oil that result in its higher antioxidant activity in sunflower oil-in-water emulsion. Another important factor that determines the efficiency of antioxidants in lipid systems is their ability to be transferred to the active site of oxidation. A highly reactive antioxidant needs to be mobile and to diffuse easily to the site of action. Viscosity is an important factor that can govern the transfer rate and mobility of antioxidants in lipid systems. A decrease in viscosity can improve the mass transfer of antioxidants in lipid systems^16^. In this study, the dynamic viscosity of sunflower oil and sunflower oil-in-water emulsion were 47.57 ± 0.70 and 2.11 ± 0.50 mPa s, respectively. The lower viscosity of sunflower oil-in-water emulsion is expected to result in a higher transfer rate of sesamol toward the actual sites of oxidation than that of sunflower oil. In addition, in this study Tween 80 was used in sunflower oil-in-water emulsion at concentration higher than its CMC value. When surfactants are used at concentration higher than their CMC value, they form micelles in the continuous phase of oil-in-water emulsions^28^. Surfactant micelles can act as carriers of lipophilic compounds, and thereby can cause an increase in the transfer rate of sesamol toward the actual site of oxidation^29^. As a consequence, the probability of effective collisions between sesamol molecules and peroxyl radicals or ROOHs in actual sites of oxidation in sunflower oil-in-water emulsion is higher than that of sunflower oil.

**Table 1:** Kinetic parameters of the initiation and propagation stages of sunflower oil oxidation.

Kinetic parameter	Bulk oil				Oil-in-water emulsion	
	Control	PGPR	Sesamol	Sesamol + PGPR	Control	Sesamol
**Initiation stage**						
IP (h)	28.80 ± 4.27^d^*	38.90 ± 6.03^ cd^	50.35 ± 0.03^ cd^	67.01 ± 1.64^a^	4.53 ± 0.31^e^	24.45 ± 0.02^d^
k_i_ × 10 (meq kg^−1^ h^−1^)	4.60 ± 0.40^c^	4.21 ± 0.08^c^	3.17 ± 0.01^d^	2.54 ± 0.05^e^	20.76 ± 0.26^a^	11.23 ± 0.27^b^
E_i_	**–**	1.38 ± 0.41^b^	1.77 ± 0.27^b^	2.36 ± 0.41^b^	–	5.27 ± 0.60^a^
ORR_i_	**–**	0.92 ± 0.06^a^	0.69 ± 0.06^b^	0.55 ± 0.06^bc^	–	0.54 ± 0.07^c^
A	**–**	1.20 ± 0.02^c^	2.34 ± 0.30^bc^	3.24 ± 0.43^b^	–	9.74 ± 0.20^a^
O_i_ (h^2^ meq^−1^ kg)	63.37 ± 4.79^d^	92.39 ± 12.67^c^	158.62 ± 0.37^b^	263.85 ± 12.03^a^	2.18 ± 0.12^d^	21.15 ± 1.41^d^
[LOOH]_IP_ (meq kg^−1^)	15.85 ± 0.74^ cd^	19.57 ± 2.89^bc^	19.16 ± 0.17^bcd^	20.27 ± 1.09^b^	16.07 ± 0.07^d^	29.85 ± 0.53^a^
**Propagation stage**						
T_max_ (h)	82.35 ± 4.26^d^	95.99 ± 3.52^c^	115.88 ± 0.16^b^	132.75 ± 2.15^a^	21.18 ± 2.05^f^	40.24 ± 0.05^e^
R_max_ (meq kg^−1^ h^−1^)	1.37 ± 0.09^c^	1.35 ± 0.10^c^	1.36 ± 0.00^c^	1.35 ± 0.01^c^	5.64 ± 0.00^a^	4.23 ± 0.04^b^
R_n_ × 10^2^ (h^−1^)	0.76 ± 0.00^c^	0.70 ± 0.01^c^	0.63 ± 0.00^c^	0.62 ± 0.01^c^	2.58 ± 0.25^a^	2.09 ± 0.04^b^
[LOOH]_Tmax_ (meq kg^−1^)	89.87 ± 5.67^b^	96.97 ± 5.27^ab^	108.89 ± 0.05^a^	109.46 ± 0.43^a^	101.30 ± 0.76^a^	110.04 ± 10.56^ab^
k_c_ × 10^2^ (h^−1^)	3.06 ± 0.01^d^	2.80 ± 0.06^d^	2.52 ± 0.00^d^	2.48 ± 0.02^d^	10.30 ± 0.99^a^	8.35 ± 0.14^b^
k_d_ × 10^4^ (meq^−1^ kg h^−1^)	1.70 ± 0.10^b^	1.44 ± 0.05^b^	1.15 ± 0.00^b^	1.13 ± 0.01^b^	4.12 ± 0.10a	4.72 ± 0.90a
PP (h)	118.98 ± 0.21^c^	128.54 ± 3.96^b^	145.02 ± 0.04^a^	146.37 ± 1.18^a^	36.17 ± 3.61^a^	39.73 ± 0.44^a^
ET_pp_ (h)	147.78 ± 4.06^d^	167.44 ± 2.07^c^	195.36 ± 0.19^b^	213.38 ± 2.81^a^	40.69 ± 3.93^f^	64.19 ± 0.46^e^
[LOOH]_max_ (meq kg^−1^)	179.74 ± 6.80^b^	193.93 ± 1.07^ab^	217.79 ± 0.09^a^	218.92 ± 0.87^a^	220.09 ± 21.11^a^	202.60 ± 1.51^ab^
E_p_	–	1.08 ± 0.03^b^	1.22 ± 0.00^ab^	1.23 ± 0.01^a^	–	1.10 ± 0.10^ab^
ORR_c_	–	0.82 ± 0.00^a^	0.92 ± 0.02^ab^	0.81 ± .00^b^	–	0.81 ± 0.06^b^
ORR_d_	–	0.85 ± 0.08^a^	0.68 ± 0.04^a^	0.67 ± 0.05^a^	–	0.89 ± 0.15^a^
IA_c_	–	1.18 ± 0.02^a^	1.48 ± 0.01^a^	1.52 ± 0.02^a^	–	1.36 ± 0.23^a^
IA_d_	–	1.27 ± 0.08^b^	1.80 ± 0.10^a^	1.85 ± 0.14^a^	–	1.11 ± 0.09^b^

In each row, means with different superscript letters are significantly different (*P* < 0.05).

*IP* induction period, *k*_*i*_ pseudo-zero order rate constant at the initiation stage, *E*_*i*_ antioxidant effectiveness during the initiation stage, *ORR*_*i*_ oxidation rate ratio during the initiation stage, *A* antioxidant activity, *O*_*i*_ oxidizability index, [*LOOH*]_*IP*_ lipid hydroperoxides concentration at IP point, *T*_*max*_ occurrence time of maximum rate of lipid hydroperoxide formation at the propagation stage, *R*_*max*_ maximum rate of lipid hydroperoxide formation in the propagation stage, *R*_*n*_ normalized form of maximum rate of lipid hydroperoxides formation in the propagation stage, [*LOOH*]_*Tmax*_ lipid hydroperoxides concentration at the point of T_max_, *k*_*c*_ pseudo-first order rate constant of LOOHs formation at the propagation stage, *k*_*d*_ pseudo-second order rate constant of LOOHs decomposition at the propagation stage, *PP* duration of the propagation stage, *ET*_*pp*_ end time of the propagation stage, [*LOOH*]_*max*_ maximum concentration of produced LOOHs, *C* integration constant, *E*_*p*_ antioxidant effectiveness during the propagation stage, *ORR*_*c*_ oxidation rate ratio of LOOHs formation during the propagation stage, *ORR*_*d*_ oxidation rate ratio of LOOHs decomposition during the propagation stage, *IA*_*c*_ inhibitory activity against the LOOHs formation, *IA*_*d*_ inhibitory activity against the LOOHs decomposition.

*Mean ± SD (*n* = 3).

**Figure 3:**
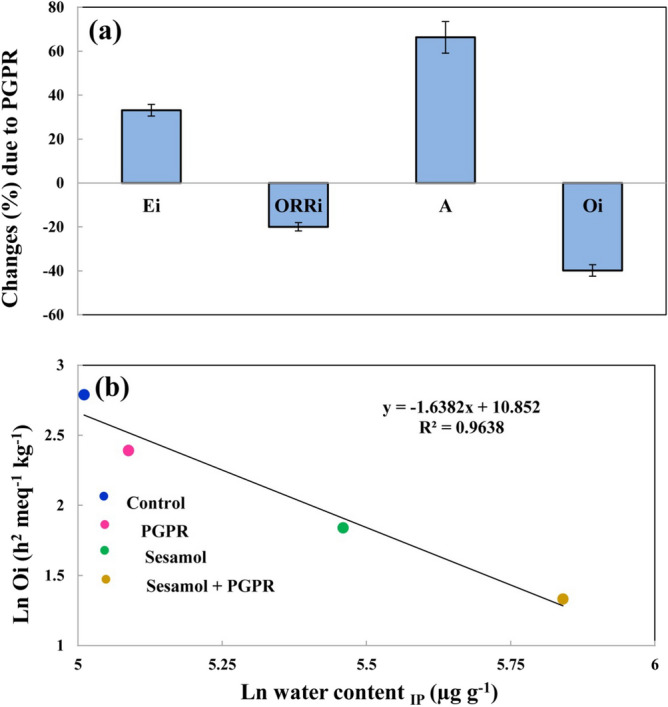
(**a**) Changes (%) in the kinetic parameters (*E*_*i*_ effectiveness of sesamol in the initiation stage, *ORR*_*i*_ oxidation rate ratio in the initiation stage, *A* antioxidant activity, *O*_*i*_ oxidizability in the initiation stage) of purified sunflower oil in the presence of polyglycerol polyricinoleate (PGPR), and (**b**) relationship between the O_i_ value of sunflower oil samples and water content at the induction period (IP) point.

PGPR was incorporated into the sunflower oil to improve the interfacial activity of sesamol in sunflower oil. Incorporating PGPR into the functional environment of sesamol resulted in a remarkable change in the mechanism of hydrogen donating of sesamol, which ultimately increased the E_i_ and A values and decreased the ORR and O_i_ values (Fig. 3a). PGPR did not show any significant antioxidant activity in the stripped sunflower oil, but synergistically improved the inhibitory effect of sesamol (SE_i_ of 41.50%). This implies the ability of PGPR in transferring sesamol to the water–oil interfaces created by reverse micelles, which results in improving the accessibility of sesamol to the reactive free radicals.

According to Fig. 3b, the O_i_ value was decreased by increasing the amount of produced water in the initiation stage of lipid oxidation. A linear relationship was found between the O_i_ value and the amount of water being produced during the initiation stage of the lipid oxidation (R^2^ = 0.964).

During the oxidation process, the water contents of sunflower oil samples were increased significantly due to the occurrence of bimolecular reactions of LOOHs (LOOH + LH → LO^·^ + L^·^ + H_2_O). In samples containing sesamol, the amounts of water increased to the higher extent than that of the control sample (Table 2). It has been shown that sesamol participates in the side reactions of chain initiation^14^. Accordingly, the higher amounts of water in samples containing sesamol can be related to the participation of a part of this molecule in side reactions of the chain initiation (AH + LOOH → LO^·^ + A^·^ + H_2_O), which produces water. The produced water plays a key role in the interfacial activity of sesamol during the oxidation process^13^.

**Table 2:** Reverse micelles size and water content of sunflower oil samples.

Sample		Reverse micelles size (× 10^–2^) (nm)				Water content (µg g^−1^)		
	BIO	BIP	IP	AIP	PP	BIO	IP	PP
Control	0.57 ± 0.05^Eb^*	5.60 ± 0.05^Cc^*	7.49 ± 0.03^Bc^	1.30 ± 0.08^Dc^	10.93 ± 0.09^Ac^	110 ± 4^Ca^	140 ± 1^Bd^	320 ± 1^Ad^
PGPR	1.572 ± 0.43^ Da^	9.72 ± 0.70^Ca^	11.24 ± 0.35^Bb^	1.38 ± 0.07^Dc^	15.32 ± 0.05^Ab^	113 ± 3^Ca^	162 ± 1^Bc^	572 ± 3^Ac^
Sesamol	0.20 ± 0.00^Ce^	6.38 ± 0.43^BCc^	12.73 ± 0.25^ABac^	2.62 ± 0.07^Cd^	17.09 ± 1.06^Aab^	113 ± 1^Ca^	235 ± 2^Bb^	600 ± 5^Ab^
Sesamol + PGPR	0.86 ± 0.04^CDd^	8.23 ± 0.84^Bc^	13.26 ± 0.89^ABb^	3.48 ± 0.09^BCd^	18.20 ± 0.78^Aa^	111 ± 5^Ca^	300 ± 3^Ba^	706 ± 6^Aa^

In each row and for each factor means with different uppercase letters are significantly different (*P* < 0.05). In each column, means with different lowercase letter are significantly different (*P* < 0.05).

*BIO* at the beginning of the oxidation, *IP* at the induction period, *AIP* after the induction period, *PP* at the propagation period.

*Mean ± SD (n = 3).

In all sunflower oil samples, the size of reverse micelles increased by increasing the amounts of water until the end of the initiation stage (Table 2). After the point of IP, a significant reduction was observed in the reverse micelles size, which can be attributed to the production of a large amount of water through bimolecular reaction (2LOOH → LOO^·^ + LO^·^ + H_2_O). The produced water can migrate to the core of the reverse micelles of sunflower oil and cause their collapse due to the volume increase^28^. During the propagation stage of lipid oxidation, the reverse micelles were regenerated and their size was increased again. In samples containing PGPR, size of the reverse micelles increased to the higher extent than those samples without PGPR. This may be due to the greater reduction of interfacial tension in the presence of PGPR. The effect of this behavior change is well observable in increasing the reverse micelles size at the IP point (Table 2). As previously mentioned, the addition of PGPR into the sunflower oil containing sesamol improved the kinetic parameters related to the initiation stage of the lipid oxidation. This can be attributed to the increase in the size of reverse micelles in the presence of PGPR, which can increase the acceptance capacity of LOOHs in these structures. Accordingly, the effective collisions between sesamol and intermediate components of oxidation can be enhanced in the presence of PGPR.

According to Table 2, increasing the size and number of reverse micelles was parallel with the amount of water produced in the system. The water molecules produced during the oxidation process can reduce the interfacial tension by attaching to the hydrophilic part of PGPR^13^. Accordingly, the water molecules can create preliminary cores and accelerate the formation of reverse micelles in the presence of PGPR. By increasing the number of the reverse micelles in the presence of PGPR, sesamol will have higher chance to locate at the oil–water interface of reverse micelles^30,31^.

### Evaluating kinetic parameters related to the propagation stage of lipid oxidation

The propagation stage was characterized by various kinetic parameters and rate constants (Table 1). The T_max_ value can use instead of IP during the propagation stage. In both sunflower oil and sunflower oil-in-water emulsion, the T_max_ value of samples treated with sesamol was significantly higher than that of control sample (*P* < 0.05). In addition, the k_c_ and k_d_ values of sunflower oil and sunflower oil-in-water emulsion samples containing sesamol were lower than that of control sample (*P* < 0.05). The R_n_ value, which is a measure of propagation oxidizability, can be taken into account as a more comprehensive kinetic parameter encompassing the values of every single kinetic parameter and the rate constant mentioned above^20^. The lower value of R_n_ indicates a higher resistance of the system against lipid oxidation. Sesamol decreased the R_n_ value of sunflower oil and sunflower oil-in-water emulsion by 17.10% and 18.99%, respectively, compared to the control sample. These results indicate that all sesamol molecules were not consumed in the initiation stage of the lipid oxidation and a part of them was remained active in the propagation stage of the lipid oxidation.

**Figure 4:**
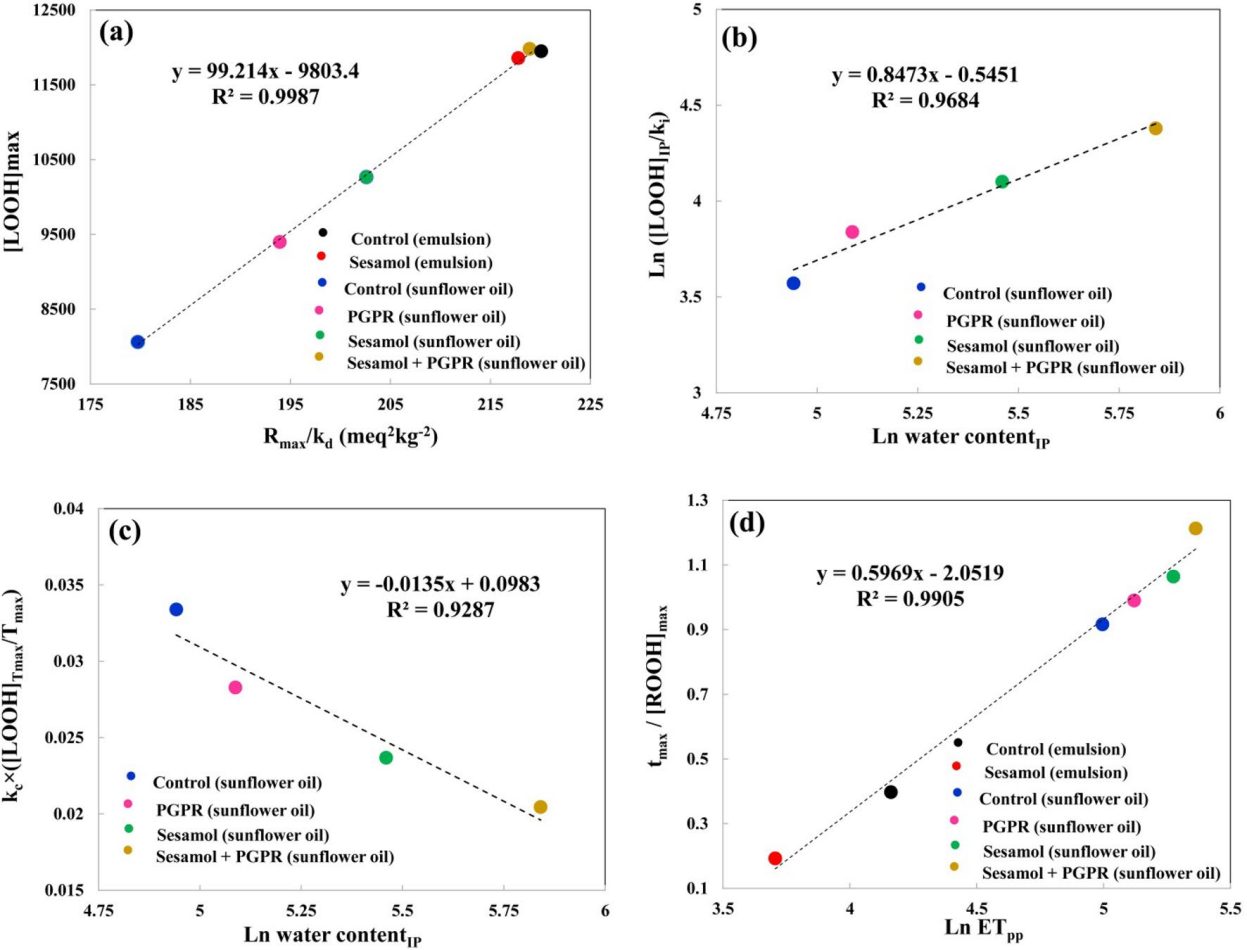
(**a,d**) Relationships between various kinetic parameters related to the propagation stage of sunflower oil and its oil-in-water emulsion samples and (**b,c**) relationships between water content at IP point and kinetic parameters related to the initiation and propagation stages of sunflower oil during peroxidation at 55 °C. *IP* induction period, *k*_*i*_ the pseudo-zero order rate constant at the initiation stage, [*LOOH*]_*IP*_ lipid hydroperoxides concentration at IP point, *T*_*max*_ occurrence time of maximum rate of lipid hydroperoxide formation at propagation stage, *R*_*max*_ maximum rate of lipid hydroperoxide formation in the propagation stage, [*LOOH*]_*Tmax*_ lipid hydroperoxides concentration at the point of T_max_, *k*_*c*_ pseudo-first order rate constant of LOOHs formation at the propagation stage, *k*_*d*_ pseudo-second order rate constant of LOOHs decomposition at the propagation stage, *ET*_*pp*_ end time of propagation stage, and [*LOOH*]_*max*_ maximum concentration of produced LOOHs.

The [LOOH]_max_ value of sunflower oil and sunflower oil-in-water emulsion is affected by the k_c_ and k_d_ values at the same time. A high correlation (R^2^ > 0.99) was found between [LOOH]_max_ value and the ratio between R_max_ and k_d_ (Fig. 4a). In both sunflower oil and sunflower oil-in-water emulsion samples, no significant difference was found between the parameter related to the inhibitory activity of sesamol against formation of LOOHs (IA_c_) with the parameter related to the inhibitory activity of sesamol against decomposition of LOOHs (IA_d_). Therefore, trend of variations in the formation and decomposition of LOOHs were completely interdependent during the propagation stage^32^.

The T_max_ value of sunflower oil sample containing combination of sesamol and PGPR was significantly higher than that of sunflower oil sample containing sesamol alone (Table 2). Accordingly, PGPR efficiently enhanced the antioxidant activity of sesamol in sunflower oil during the propagation stage (SE_p_ of 36.84%) as in the initiation stage (SE_i_ of 68.87%). This can be related to the physical role of PGPR on inhibiting the oxidation reactions. Surface-active LOOH molecules produced during the propagation stage of the lipid oxidation have a high affinity to migrate toward the reverse micelles^27^. During the propagation stage, the size of reverse micelles in samples containing sesamol + PGPR was significantly higher than those samples containing sesamol alone (Table 2). Accordingly, the acceptance capacity of LOOHs in the reveres micelles was increased in the presence of PGPR, which resulted in higher accessibility of sesamol to LOOHs. This physical role of PGPR can postpone the occurrence of turning point in the propagation stage.

The water produced in sunflower oil during the lipid oxidation process exhibited various linear relationships with some kinetic parameters related to the initiation or propagation stage. A high correlation (R^2^ = 0.944) was found between water content at IP point and the ratio between LOOH_IP_ and k_1_ (Fig. 4b). This indicates that enhancing the amount of water during the initiation stage reduced the lipid oxidation rate at this stage. In addition, a high correlation between the kinetic parameters related to the propagation stage (k_f_ × ([LOOH]_Tmax_/Tmax) and water content at IP points indicates that the water produced during the initiation stage can alter the lipid oxidation rate at the propagation stage (Fig. 4c). According to Fig. 4d, a high correlation exists between the end time of the propagation stage and the ratio of occurrence time of turning point and LOOHs concentration at the turning point (T_max_/[LOOH]_Tmax_). This reveals that postponing the occurrence time of maximum rate of LOOHs formation can enhance the duration of the propagation period.

## Conclusion

In this study, antioxidant activity of sesamol in sunflower oil and its oil-in-water emulsion was investigated over the whole practical range of peroxidation, including the initiation and propagation stages. In both sunflower oil and sunflower oil-in-water emulsion, the inhibitory effect of sesamol was not limited to the initiation stage of lipid oxidation and a part of this molecule was also active in the propagation stage. Sesamol exhibited better antioxidant performance in sunflower oil-in-water emulsion than that of sunflower oil. This was attributed to the higher interfacial activity of sesamol in sunflower oil-in-water emulsion than that of sunflower oil. In addition, the lower viscosity of sunflower oil-in-water emulsion than that of sunflower oil indicates the higher transfer rate of sesamol toward the actual sites of oxidation in sunflower oil-in-water emulsion than that of sunflower oil. PGPR was incorporated into sunflower oil to enhance the interfacial performance of sesamol in sunflower oil. Incorporating PGPR into the functional environment of sesamol lead to a significant change in the mechanism of hydrogen donating of sesamol which ultimately improved the kinetic parameters related to the initiation stage and propagation stage. Comparison of the size of reverse micelles in samples containing PGPR with those without PGPR showed that PGPR can improve the antioxidant performance of sesamol by increasing the acceptance capacity of LOOHs in reveres micelles structures. Therefore, the effective collision between sesamol and LOOHs is expected to increase in the presence of PGPR. The water produced as a major byproduct of lipid oxidation played a key role on the efficiency of sesamol alone or in combination with PGPR during the initiation stage of lipid oxidation. In general, sesamol exhibits better interfacial activity in sunflower oil-in-water emulsion than that of sunflower oil. In addition, PGPR can improve the interfacial activity of sesamol in sunflower oil. The results of this study can help manufacturers of food industry to reduce lipid oxidation by using the most adapted antioxidative strategies for their specific products.

## Data Availability

The data used to support the findings of this study are included within the article.
